# Air versus fluorinated gas tamponades in pars plana vitrectomy treatment for primary rhegmatogenous retinal detachment

**DOI:** 10.1111/aos.15144

**Published:** 2022-03-29

**Authors:** Birgit Marlies Govers, Martijn P.M. Lamers, B. Jeroen Klevering, Sander Keijser

**Affiliations:** ^1^ Department of Ophthalmology Radboud University Medical Centre Nijmegen The Netherlands

**Keywords:** air tamponade, fluorinated gas tamponade, inferiorly located retinal detachments, inferiorly located retinal tears, pars plana vitrectomy, Rhegmatogenous retinal detachment

## Abstract

**Purpose:**

To compare the treatment success of air with fluorinated gas (20% SF_6_ or 14% C_3_F_8_) tamponade in pars plana vitrectomy for primary rhegmatogenous retinal detachment.

**Methods:**

A retrospective cohort study comprised of 1023 consecutive primary retinal detachment cases between 2014 and 2020. We employed a univariate multivariable binary logistic regression model.

**Results:**

We used intraocular gas tamponades in 872 cases with PVR grade B or lower: air tamponade was used in 414 eyes and 458 eyes were treated with a type of fluorinated gas tamponade. There was no significant difference in the type of tamponade with regard to the re‐detachment rate (95% CI −1.0% and 4.1%). Additionally, also in the subgroup of rhegmatogenous retinal detachments with inferior located retinal defects we found no significant difference between the two types of tamponade (p = 0.54 Fisher's exact). The multivariable model, which included tamponade, PVR grade, a retinal detachment involving the 6 o'clock position and age as covariates, also showed no significant effect of tamponade choice on treatment success (OR 0.5, 95% 0.2–1.0, p = 0.10).

**Conclusion:**

We found no difference in treatment success with air tamponade versus fluorinated gas tamponades in the repair of primary retinal detachments, this also includes inferiorly located retinal tears and detachments.

## Introduction

Pars plana vitrectomy (PPV) with an intraocular gas tamponade is the most common used approach to achieve retinal reattachment in primary rhegmatogenous retinal detachments (RRDs) (Poulsen et al. 2016; Nemet et al. 2017; Neffendorf et al. 2018; Kunikata et al. 2019). Frequently used tamponade agents include air, SF_6_ and C_3_F_8_ gas (Poulsen et al. 2016; Vaziri et al. 2016; Neffendorf et al. 2018). In vitreoretinal surgery, concentrations of 20% SF_6_ and 14% C_3_F_8_ are used for completely filling the vitreous cavity because pure SF_6_ and C_3_F_8_ gas may double and quadruple in volume as result of nitrogen and oxygen diffusion in to the vitreous cavity. By contrast, air is non‐expansile (Chan et al. 2008; Vaziri et al. 2016). Although all gas tamponades absorb spontaneously from the vitreous cavity, the half‐life of these gas tamponades varies greatly (Thompson 1989; Mateo‐Montoya & de Smet 2014; Poulsen et al. 2016; Vaziri et al. 2016). Air tamponade disappears after approximately 7–10 days, this can take 2–3 weeks in case of 20% SF_6_ and up to 8 weeks with a 14% C_3_F_8_ gas tamponade (Mateo‐Montoya & de Smet 2014; Vaziri et al. 2016; Chen et al. 2018; Neffendorf et al. 2018).

The high surface tension of a gas bubble prevents fluid flow through a retinal defect while retina‐RPE adhesion is still weak (Vaziri et al. 2016; Neffendorf et al. 2018). This allows the active and passive fluid transport across the RPE to re‐attach the neuroretina (Marmor 1990). Firm retina‐RPE adhesion is further enhanced by photo‐ or cryocoagulation and the subsequent formation of a chorioretinal scar (Ang et al. 2012). Experimental research has shown that retina‐RPE adhesion induced by photocoagulation exceeds regular adhesion after 24 hr (Yoon & Marmor 1988). In view of this time scale, we hypothesize that treatment success of air tamponade should be comparable to longer acting gas tamponades, under the condition that centripetal forces caused by vitreous and subretinal fluid are sufficiently removed during PPV (Martinez‐Castillo et al. 2005; Mateo‐Montoya & de Smet 2014; Pak et al. 2017; Lin et al. 2018).

The use of air tamponade has potential several benefits: it enables faster visual recovery due to its shorter half‐life, a possibly earlier recognition of retinal re‐detachment, carries a diminished risk of postoperative elevated intraocular pressure and may cause less progression to cataract development (Zhou et al. 2015; Pak et al. 2017; Li et al. 2020). In addition, air is cheap and has no environmental impact, unlike fluorinated gases (Rabie & Franck 2018). Air tamponade provides initial reattachment rates of 93% or more in primary RRDs (Martinez‐Castillo et al. 2005; Mateo‐Montoya & de Smet 2014; Li et al. 2020). Two studies showed equivalent treatment success in a comparison of gas tamponade groups (Zhou et al. 2015; Pak et al. 2017). Still, it is debated whether short‐acting gas tamponades are effective in tamponing inferior located retinal tears (Heimann et al. 2006; Kunikata et al. 2019). A large retrospective study comparing 20% SF_6_ and air tamponades reported more favourable outcomes with fluorinated gas tamponade for inferior located detachments (Tan et al. 2013).

In the Radboud University Medical Centre, Nijmegen, the use of 20% SF_6_ and 14% C_3_F_8_ gas has gradually been replaced by air tamponade between 2014 and 2020. We compared treatment results of air with fluorinated gas tamponades in PPV treatment of primary RRDs. We specifically analysed whether the choice of tamponade affected treatment success in inferiorly located retinal tears and inferiorly located retinal detachments.

## Methods

### Overview

This study was conducted at the referral centre for vitreoretinal surgery at the Radboud University Medical Centre, Nijmegen, the Netherlands. For this retrospective cohort study, we included all patients with a primary RRD that underwent a PPV with air, 20% SF_6_ or 14% C_3_F_8_ tamponade between June 2014 and May 2020. All patients were operated by one experienced surgeon (SK) and the minimal follow‐up was 6 months. The decision to use either air or fluorinated gas tamponade was initially based on position of the breaks: only retinal detachments with a superior break were treated with air tamponade. This gradually extended to the use of air tamponade in all cases, regardless location of retinal breaks, during the last year of study period. We did not include cases in which a recurrent retinal detachment was treated or in which silicone oil tamponade was used. We excluded RRDs with a PVR grade C. This study was conducted in accordance with the principles of the Declaration of Helsinki and was carried out in accordance with the applicable legislation concerning review by the research ethics committee Arnhem‐Nijmegen.

### Outcome measurements

We defined primary treatment success as an attached retina without retinal re‐detachment within 6 months after surgery. Final anatomical success was defined as an attached retina in the absence of a tamponade agent *in situ* at the last recorded follow‐up examination.

We expressed the location of the retinal defects and the area of retinal detachment in clock hours and defined an inferior location separately for defects and detachments. According to our definition, an inferiorly retinal defect was located at clock hours 4, 5, 6, 7 and/or 8, and an inferior retinal detachment involved clock hours 4, 5, 6, 7 and/or 8.

Proliferative vitreoretinopathy (PVR) was classified according to the Updated Retina Society Classification (Machemer et al. 1991). We combined ‘no PVR’ and ‘PVR grade A’ in one group, given the minimal clinical differences.

### Surgical procedure

For the vitrectomy, we used an Alcon Constellation platform with 23 gauge or 25 gauge (after 2015) trocars. We used a Zeiss (Oberkochen, Germany) Resight® or Volk® A.V.I. lens panoramic viewing system for visualization of the retina. Following core vitrectomy, the peripheral vitreous was stained with triamcinolone (Kenacort®; Bristol‐Myers Squibb, New York, NY, USA) and the vitreous based was shaved while indentation was established with a 25‐gauge light pipe. A second round with staining and shaving was performed to scrupulously remove most vitreous as possible. After meticulous removal of the vitreous, the retina was attached by draining subretinal fluid under air. We used perfluoro‐decalin liquid (DK‐line®; Bausch + Lomb, Bridgewater, NJ, USA) if there was persisting subretinal fluid, which threatened to create a macular fold or in persistent bullous retinal detachments. The inner limiting membrane (ILM) was only peeled when there was a macular epiretinal membrane or wrinkling of the ILM present during surgery. We treated all retinal defect with photocoagulation; in almost all cases, we added a 360 degrees band of peripheral prophylactic laser. A 360 degrees laser band was applied to close potential iatrogenic retinal holes created during precise vitreous removal at the vitreous base, as we strive to remove as much vitreous as possible to reduce the risk of renewed vitreous traction. Immediately after surgery, after the infuse port was removed, the eye globe was brought to tension with an additional intravitreal injection of the tamponade that was used. Sclerotomies were usually not sutured because air or fluorinated gas leakage was avoided by the use of self‐sealing sclerotomies.

Patients are generally instructed as following for postoperative positioning: patients with a break inferiorly are instructed to lie on the opposite side as the break is located during three consecutive nights after surgery; patients with a detachment through the macula are instructed to lie on the temporal side for 45 min immediately after surgery, to reduce the likelihood of macular folds. No posturing instructions were given to patients with superior breaks.

### Statistical analyses

IBM spss Statistics 25.0 software (IBM Corp., Armonk, NY, USA) was used for data analyses with statistical significance defined at a p‐value of ≤0.05. Numerical data were tested with a Mann–Whitney *U* test and age with an independent samples *t*‐test. Chi‐square (*χ* ^2^) and Fisher's exact tests were performed to compare categorical data. We built a univariate multivariable binary logistic regression model to analyse the effect of tamponade choice on treatment success. The model‐building process was executed using the ‘purposeful selection method’ approach (Bursac et al. 2008). All parameters showing a univariable significant association with treatment success were included as candidate variable in the univariate multivariable model. These variables were further selected based on significance level and tested for confounding.

## Results

Between June 2014 and May 2020 1263 eyes of 1176 patients underwent RRD PPV surgery by SK at the vitreoretinal referral centre of the Radboud University Medical Centre. We included 910 eyes of 898 patients who met the inclusion criteria and underwent PPV for a primary RRD with air or fluorinated gas tamponade. We excluded 27 eyes with PVR grade C and 11 eyes of 11 patients due to objection to the use of their medical data. This resulted in a total of 872 eyes of 861 patients treated for a primary RRD.

### Intraocular tamponades

**Fig. 1:**
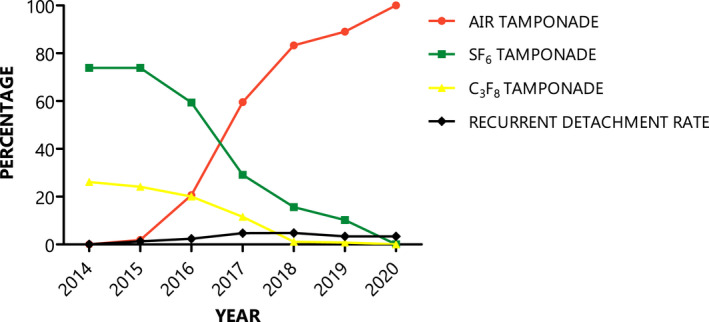
Type of tamponade and recurrent detachment rate per year.

Intraocular gas tamponades used in 872 pars plana vitrectomies indicated for primary RRD with a PVR grade B or less were: air tamponade in 414 eyes (47.5%), 20% SF_6_ gas tamponade in 349 eyes (40.0%), and 14% C_3_F_8_ gas tamponade in 109 eyes (12.5%). During the first 7 months of study period, all gas tamponades were fluorinated gas, and in the last 6 months of study period, all gas tamponades were air tamponade. Figure 1 shows proportional tamponade usage and retinal re‐detachment rate per year. The use of SF_6_ and C_3_F_8_ gas tamponade decreased in favour of air while re‐detachment rate remained similar over these years.

### Gas tamponades

#### Clinical characteristics in gas tamponade groups

Table 1 shows clinical characteristics per tamponade group. Especially early in the transition from fluorinated gas to air, we reserved air tamponade for relatively easy cases. On average, air tamponade was employed in cases with a less extensive region of detached retina, a smaller area with retinal defects, and less frequently inferior located retinal defects and inferior located retinal detachments compared to other gas tamponades. Pre‐operative PVR grade and ILM peeling were similar between tamponade groups. Primary treatment success was achieved in 96.9% (*n* = 845). The type of tamponade did not significantly influence the rate of retinal re‐detachment (risk difference 95% confidence interval −1.0% to 4.1%). Additionally, we found no difference in treatment success between tamponade groups in the subgroup of RRDs with retinal defects or detachments located inferiorly (p = 0.54 Fisher's exact, p = 0.47 Chi‐square). The more specific subgroup of RRDs with retinal defects or detachment involving the 6 o'clock position also showed no difference in treatment success between tamponade groups (p = 0.39 Fisher's exact, p = 0.17 Chi‐square). Three quarters of the retinal re‐detachments (74.1%) occurred within 50 days after the surgical intervention. A re‐detachment developed significantly earlier in patients treated with air compared to other gas tamponades: median 22 versus 46 days (p = 0.002, Mann–Whitney *U*). In 18 re‐detachments (66.7%) one additional procedure, and in 9 (33.3%) cases, two additional pars plana vitrectomies were necessary to achieve a permanently attached retina. We achieved a final anatomical treatment success in 99.7% (*n* = 869) of all RRD patients initially treated with vitrectomy and some type of gas tamponade. In three patients, who did not meet the final treatment success criteria, we did manage to attach the retina albeit under permanent silicone oil tamponade.

**Table 1:** Clinical‐ and treatment characteristics of primary rhegmatogenous retinal detachments treated by pars plana vitrectomy with air or fluorinated gas tamponade.

	Data expressed as	Air	SF_6_ and C_3_F_8_ gas	p
Air: SF_6:_ C_3_F_8_	*n*=	414: 0: 0	0: 349: 109	
Age (years)	Mean, SD	62, 9	61, 10	*0.008*
Male sex	% (*n*=)	69.6 (288)	65.9 (302)	0.25
Lens status				
Phakic	% (*n*=)	67.4 (279)	64.1 (293)	0.58
Pseudophakia	% (*n*=)	31.9 (132)	35.2 (161)	
Aphakic	% (*n*=)	0.7 (3)	0.7 (3)	
Foveal involvement				
Fovea attached	% (*n*=)	57.8 (237)	55.5 (251)	0.50
Fovea detached	% (*n*=)	42.2 (173)	44.5 (201)	
Size retinal detachment (clock hours)	Mean, median (Q1–Q3)	5.3, 5.0 (4.0–6.0)	5.6, 5.0 (4.0–7.0)	*0.050*
Area with retinal defects (clock hours)	Mean, median (Q1–Q3)	1.7, 1.0 (1.0–2.0)	2.4, 2.0 (1.0–3.0)	*<0.001*
Inferior located retinal detachment^†^	% (*n*=)	64.6 (267)	73.6 (331)	*0.005*
Retinal detachment in 6 o'clock	% (*n*=)	22.5 (93)	40.0 (180)	*<0.001*
Inferior located retinal defect^‡^	% (*n*=)	22.0 (90)	52.7 (240)	*<0.001*
Retinal defect in 6 o'clock	% (*n*=)	3.9 (16)	17.6 (80)	*<0.001*
PVR grade				
No PVR or PVR grade A	% (*n*=)	68.1 (282)	64.0 (288)	0.20
PVR grade B	% (*n*=)	31.9 (132)	36.0 (162)	
ILM peeling	% (*n*=)	2.7 (11)	3.9 (18)	0.30
Re‐detachment after treatment	% (*n*=)	3.9 (16)	2.4 (11)	0.213
Days until re‐detachment	Mean, median (Q1–Q3)	32, 22 (15–36)	68, 46 (38–131)	*0.002*
Final anatomical success	% (*n*=)	99.5 (411)	100.0 (458)	0.225

Statistically significant values are represented in italics.

ILM = inner limiting membrane, PVR = proliferative vitreoretinopathy.

^†^ An inferior located retinal detachment was defined as a detachment involving clock hours 4, 5, 6, 7 and/or 8.

^‡^ An inferior located retinal tear was defined as a defect involving clock hours 4, 5, 6, 7 and/or 8.

The gradual transition in choice of tamponade based on the involvement of retinal defects and detachments inferiorly are shown per year in Table S1. Each subsequent year a higher percentage of inferior tears and inferior retinal detachments were treated with air tamponade.

We compared clinical characteristics and treatment success between tamponade groups while using only cases of the first seven and last 6 months of study period, that is the periods in which only fluorinated gas and only air tamponade were used respectively (Table S2). This small cohort showed no difference in the extent of detached retina, the size of area with retinal defects, inferiorly located retinal detachments or defects and retinal detachments or tears involving the 6 o' clock position between tamponade groups. We found no difference in treatment success between tamponade groups in these cases.

#### Associations with treatment success

To select candidate variables for the univariate multivariable binary logistic regression model, we first univariably compared clinical characteristics of primary successful (*n* = 845) and unsuccessful (*n* = 27) treatment (Table S3). Treatment success was not associated with inferiorly located retinal tears, inferior located retinal detachments or wider distribution of the retinal defects (p = 0.60, p = 0.33, p = 0.62 respectively). Factors that negatively influenced treatment outcome were: higher age, a larger area of detached retina and higher pre‐operative PVR grades. We observed a trend towards more frequent ILM peeling in retinal detachments with unsuccessful primary treatment outcome. Although inferior located retinal detachments were not associated with worse outcomes, we did find an association for retinal detachments that specifically did or did not involve the 6 o'clock position. In 273 cases, the RRD included this most inferior part of the retina and in 5.9% (*n* = 16) a retinal re‐detachment occurred, this compared to 1.9% (*n* = 11) in the 590 RRD that did not involve the 6 o'clock position (*χ* ^2^, p = 0.002). The factors associated with treatment success were tested for correlations. The extent of detached retina strongly associated with two important parameters related to treatment success: increasing PVR grade (p < 0.001) and detachments involving the 6 o'clock position with median 7 (Q_1_–Q_3_; 6–9) versus 5 (Q_1_–Q_3_; 4–6) clock hours respectively (p < 0.001). In addition, PVR was more severe in detachments involving clock hour 6: PVR grade B was observed in 43.2% (*n* = 118) detachments reaching the 6 o'clock position, compared to 29.6% (*n* = 172) in the remaining RRDs (*χ* ^2^, p < 0.001).

We built a univariate multivariable binary logistic regression model to analyse the effect of tamponade choice on treatment success. Candidate variables were the type of tamponade (air versus fluorinated gas) and all parameters that were univariably associated with treatment success. The final multivariable model included tamponade, PVR grade, retinal detachment involving clock hour 6 and age as covariates. This model did not show a significant difference in air versus fluorinated gas on treatment success (OR 0.5, 95% 0.2–1.0, p = 0.10; Table 2).

**Table 2:** Univariate multivariable logistic regression model for treatment success.

	*β*	OR	95% CI	p
Air tamponade (SF_6_ and C_3_F_8_ gas reference)	−0.68	0.5	0.20–1.0	0.10
No PVR/PVR grade A (PVR grade B reference)	0.71	2.0	0.92–4.5	0.08
Retinal detachment not involving 6'o clock (involving 6'o clock reference)	1.10	3.0	1.3–6.9	0.01
Age	−0.05	0.95	0.91–0.99	0.02

Data set variable coding: treatment success (0 = unsuccessful primary treatment, 1 = successful primary treatment), tamponade (0 = fluorinated gas, 1 = air), PVR grade (0 = PVR grade B, 1 = no PVR/PVR A), retinal detachment location (0 = detachment involving 6'o clock position, 1 = detachment not involving 6'o clock position), age (age at retinal detachment in years).

CI = confidence interval, OR = odds ratio, PVR = proliferative vitreoretinopathy.

We calculated re‐detachment risk per extent of detached retina in cases with any type of gas tamponade. The re‐detachment rate of 142 eyes with 1–3 clock hours of detached retina was 1.4% (*n* = 2), this risk gradually increased to 2.5% (*n* = 12) in 487 cases with 4–6 clock hours of detachment, 4.4% (*n* = 8) in 182 cases with 7–9 clock hours, and finally 9.6% (*n* = 5) in 52 cases with 10–12 clock hours of retinal detachment.

## Discussion

### General

In the period 2014–2020, we gained experience with air tamponade and increasingly used air in lieu of SF_6_ or C_3_F_8_ gas. Air tamponade enhances postoperative recovery and reduces the costs of surgery. Moreover, sulphur hexafluoride is a strong greenhouse gas, which use should be discouraged unless absolutely necessary (Rabie & Franck 2018). Nowadays, we also use air tamponades in all primary RRDs with inferiorly located retinal tears and inferiorly located retinal detachments with PVR grade A or B. Currently, there is no convincing evidence to either support or negate the use of air tamponade in inferior RRDs. Previous studies either excluded inferiorly located retinal tears or inferiorly located retinal detachments from their analyses, were limited by small patient cohorts, or made no comparison between the type of gas tamponades (Martinez‐Castillo et al. 2005; Tan et al. 2013; Mateo‐Montoya & de Smet 2014; Zhou et al. 2015; Pak et al. 2017; Chen et al. 2018; Lin et al. 2018; Li et al. 2020). This study compromises a large cohort of 872 eyes with primary RRD. We show a relative high treatment success in these 872 cases with a PVR grade B or less where gas tamponade was used. We found no difference in treatment success with air tamponade versus fluorinated gas tamponades in the repair of primary retinal detachments, this also includes inferiorly located retinal tears and detachments and more specifically retinal tears and detachments involving the 6 o'clock position. Earlier research indicated that the success rate of air tamponade was significantly lower compared to fluorinated gas tamponades for retinal detachments involving inferior quadrants (Tan et al. 2013). However, while comparing treatment success of tamponades in inferior retinal detachments, Tan et al. (2013) only selected specific cases with retinal defects located non‐inferiorly. The exclusion of all inferior located retinal defects from their cohort may have led to selection bias. In agreement with previously definitions, we classified ‘inferior’ retinal detachment, as any detached retina between 4–8 clock hours. This arbitrary cut‐off value potentially introduces bias, because our analysis shows treatment success is strongly affected by a more specific parameter: the absence or presence of retinal detachment at the 6 o' clock position. The significantly higher re‐detachment risk in these retinal detachments, was not influenced by the type of gas tamponade and thus, may simply reflect a difference in PVR grade or insufficient short term tamponade by both air and fluorinated gas. PVR grade can, at least partially, explain the lower treatment success in detachments at the 6 o'clock position. We can back this hypothesis up with two arguments. First, retinal detachment involving 6 o'clock had significantly more severe PVR and larger detachments. Second, removal of PVR grade as covariate in the multivariable treatment success model increased effect size of the involvement of clock hour 6 in detachments. The more severe PVR in detachments involving the 6 o'clock position is likely driven by the longer duration of retinal detachments since these typically show a late presentation.

The extent of detached retina was univariable significantly associated with treatment success. Nevertheless, the extent of retinal detachment showed no significant association in the multivariable model and was not a confounding factor in this model. In contrast to our results, the amount of detached quadrants was found independently related to treatment success in a previous study (Zhou et al. 2015). This discrepancy might be induced by the categorical use of detachment per each of four quadrants involved, instead of using data per clock hour of retinal detachment. Baseline differences in PVR grade may also have interfered the relation between the extent of detached retina and treatment success: only PVR grade B and C1 were included (Zhou et al. 2015). We hypothesize that the association between size of detachment and treatment success was almost completely expressed in PVR grade. A larger area of detachment conceivably induces a stronger inflammatory response, as result of blood‐retinal barrier disruption and retinal hypoxia, ultimately leading to the development of PVR (Idrees et al. 2019). In this cohort, the re‐detachment risk was 2.5 higher in PVR grade B compared to lower PVR grades. The association between proliferative vitreoretinopathy development and treatment success is also well established (Idrees et al. 2019). Since the extent of detached retina was strongly associated with PVR grade, and, since it is an easily interpretable and more externally valid parameter, we calculated re‐detachment risks per three continuous clock hours of detached retina. Future research might explore whether there is a critical extent of detached retina in which silicone oil tamponade might be preferred over air/gas tamponades.

Age showed a highly significant, though moderate, association with primary anatomical success in our cohort. Increasing age could be associated with longer patient's delay, which leads to higher PVR grades. Removal of age as covariate in the multivariable model increased PVR grade's effect size. The trend towards more re‐detachments in cases where the ILM was peeled, probably reflects the surgeon's choice of peeling this membrane in cases with a more severe degree of PVR during initial surgery.

Postoperative positioning could be an important factor for treatment success in inferior detached cases. Patients compliance remains an uncertain factor, though this mostly will affect the fluorinated gas group since, on average, these had more frequently inferior located retinal detachments. Nevertheless, comparing tamponade groups while using only cases of the first seven and last 6 months of study period, in other words, the months without selection bias in gas tamponade choice, also showed no difference in treatment success Although these data must be interpreted with caution due to the relatively small sample size, the tamponades groups were similar in terms of inferiorly located retinal detachments or defects, and retinal detachments or defects involving the 6 o'clock position during this period.

Time period until a re‐detachment developed was significantly shorter in air tamponade compared to other gas tamponades: 22 versus 46 days median. Earlier research reported similar findings: on average 9 versus 21 days respectively (Pak et al. 2017). However, in our cohort, duration until re‐detachment far exceeds the expected tamponade duration: air is completely resolved from the vitreous cavity after 6–11 days, and this is 2 and 8 weeks using 20% SF_6_ and 14% C_3_F_8_ respectively (Mateo‐Montoya & de Smet 2014; Vaziri et al. 2016; Chen et al. 2018; Neffendorf et al. 2018). The shorter time period until re‐detachment occurs after air tamponade might, therefore, not correspond to an earlier recognition of recurrent detachment compared to fluorinated gas tamponade, and thus may not affect treatment outcome of secondary interventions.

### Limitations

Tamponade groups are, inevitably related to a retrospective design, prone to selection bias. The vitreoretinal surgeon was, at least initially, less likely to use air in retinal detachments with inferior located retinal defects and inferior located retinal detachments. Nevertheless, in the later stages of this study, selection bias was no longer present. At that time the air tamponade had completely replaced the use of fluorinated gas. It is possible that the increase in experience of the surgeon during the study period partially affected the outcome. However, the surgeon was already experienced at the beginning of study period, and we, therefore, assume this had no significant effect on the outcome in this study. We restricted our cohort to patients treated by a single vitreoretinal surgeon to reduce potential confounding factors related to differences in, for example tamponade choice or treatment success between vitreoretinal surgeons.

## Supporting information


**Table S1.** The type of gas tamponade use per clinical characteristic shown per year.

**Table S2.** Clinical‐ and treatment characteristics of primary rhegmatogenous retinal detachments treated by pars plana vitrectomy with air or fluorinated gas tamponade during the first and last 6 months of study period.

**Table S3.** Clinical‐ and treatment characteristics of primary rhegmatogenous retinal detachments treated by pars plana vitrectomy with a primary successful or unsuccessful treatment.
