# Supplementary material for: Air versus fluorinated gas tamponades in pars plana vitrectomy treatment for primary rhegmatogenous retinal detachment
Source: Acta Ophthalmol. 2022 Mar 29;100(8):e1600–5. doi: 10.1111/aos.15144 (PMC9790619; doi:10.1111/aos.15144)
Supplement: Supplementary file 1 — Table S1. The type of gas tamponade use per clinical characteristic shown per year. [file AOS-100-e1600-s003.docx]

| **Supplemental Table S1. The type of gas tamponade use per clinical characteristic shown per year. All data are expressed as ‘proportion air tamponade / proportion fluorinated gas tamponade (total number of eyes)’.** | | | | | | | | |
| --- | --- | --- | --- | --- | --- | --- | --- | --- |
|  | **2014** | **2015** | **2016** | **2017** | **2018** | **2019** | **2020** | **Total** |
| **All RRDs** | 0/100 (69) | 2/98 (157) | 21/79 (165) | 59/41 (148) | 83/17 (186) | 89/11 (118) | 100/0 (29) | 47/53 (872) |
| **Inferior located retinal detachment*** | 0/100 (34) | 0/100 (103) | 12/88 (111) | 51/49 (112) | 79/21 (132) | 85/15 (89) | 100/0 (17) | 45/55 (598) |
| **Retinal detachment in 6 o’clock** | 0/100 (16) | 0/100 (43) | 3/97 (59) | 27/73 (51) | 67/33 (52) | 77/23 (43) | 100/0 (9) | 34/66 (273) |
| **Inferior located retinal defect**† | 0/100 (24) | 0/100 (66) | 5/95 (75) | 18/82 (55) | 57/43 (53) | 78/22 (49) | 100/0 (8) | 27/73 (330) |
| **Retinal defect in 6 o’clock** | 0/100 (6) | 0/100 (15) | 4/96 (26) | 11/89 (19) | 36/64 (14) | 50/50 (16) | -/- (0) | 17/83 (96) |
| **Re-detachment after treatment** | -/- (0) | 0/100 (2) | 25/75 (4) | 43/57 (7) | 89/11 (9) | 75/25 (4) | 100/0 (1) | 59/41 (27) |
| *Data are expressed as proportion air tamponade / proportion fluorinated gas tamponade (total number of eyes)*  ** An inferior located retinal detachment was defined as a detachment involving clock hours 4, 5, 6, 7, and/or 8.* †*An inferior located retinal tear was defined as a defect involving clock hours 4, 5, 6, 7, and/or 8.* | | | | | | | | |
