# Supplementary material for: Air versus fluorinated gas tamponades in pars plana vitrectomy treatment for primary rhegmatogenous retinal detachment
Source: Acta Ophthalmol. 2022 Mar 29;100(8):e1600–5. doi: 10.1111/aos.15144 (PMC9790619; doi:10.1111/aos.15144)
Supplement: Supplementary file 2 — Table S2. Clinical‐ and treatment characteristics of primary rhegmatogenous retinal detachments treated by pars plana vitrectomy with air or fluorinated gas tamponade during the first and last 6 months of study period. [file AOS-100-e1600-s002.docx]

| **Supplemental Table S2. Clinical- and treatment characteristics of primary rhegmatogenous retinal detachments treated by pars plana vitrectomy with air or fluorinated gas tamponade during the first and last six months of study period.** | | | | |
| --- | --- | --- | --- | --- |
|  | **Data expressed as** | **First half year study period ^p1^** | **Last half year study period ^p2^** | **P** |
| **Tamponade** Air SF_6_  C_3_F_8_ | *% n= % n= % n=* | 0.0 (0) 74.3 (52) 25.7 (18) | 100.0 (37) 0.0 (0) 0.0 (0) |  |
| **Age** (years) | *Mean, SD* | 60, 9 | 65, 10 | *0.01* |
| **Male** | *% (n=)* | 67.1 (47) | 64.9 (24) | 0.81 |
| **Lens status** Phakic Pseudophakia | *% (n=) % (n=)* | 67.1 (47) 32.9 (23) | 56.8 (21) 43.2 (16) | 0.29 |
| **Foveal involvement** Fovea attached Fovea detached | *% (n=) % (n=)* | 55.9 (38) 44.1 (30) | 45.9% (17) 54.1 (20) | 0.33 |
| **Size retinal detachment** (clock hours) | *Mean, median (Q1-Q3)* | Mean 5.5, median 5.0 (4.0-7.0) | Mean 5.9 median 6.0 (5.0-7.0) | 0.13 |
| **Area with retinal defects** (clock hours) | *Mean, median (Q1-Q3)* | Mean 2.1, median 2.0 (1.0-3.0) | Mean 2.0, median 1.0 (1.0-3.0) | 0.60 |
| **Inferior located retinal detachment*** | *% (n=)* | 54.0% (34) | 64.9% (24) | 0.29 |
| **Retinal detachment in 6 o’clock** | *% (n=)* | 25.4% (16) | 35.1% (13) | 0.30 |
| **Inferior located retinal defect**† | *% (n=)* | 35.3% (24) | 32.4% (12) | 0.77 |
| **Retinal defect in 6 o’clock** | *% (n=)* | 8.8% (6) | 8.1% (3) | 0.90 |
| **PVR grade** No PVR or PVR grade A PVR grade B | *% (n=) % (n=)* | 70.1 (47) 29.9 (20) | 56.8 (21) 43.2 (16) | 0.17 |
| **Re-detachment after treatment** | *% (n=)* | 0.0% (0) | 2.7% (1) | 0.35 |
| *Statistically significant values are represented in italics. ^p1^ First half year study period is between June 2014 and January 2015  ^p2^ Last half year study period is between November 2019 and May 2020 * An inferior located retinal detachment was defined as a detachment involving clock hours 4, 5, 6, 7, and/or 8. † An inferior located retinal tear was defined as a defect involving clock hours 4, 5, 6, 7, and/or 8. PVR, proliferative vitreoretinopathy.* | | | | |
