# Supplementary material for: Air versus fluorinated gas tamponades in pars plana vitrectomy treatment for primary rhegmatogenous retinal detachment
Source: Acta Ophthalmol. 2022 Mar 29;100(8):e1600–5. doi: 10.1111/aos.15144 (PMC9790619; doi:10.1111/aos.15144)
Supplement: Supplementary file 3 — Table S3. Clinical‐ and treatment characteristics of primary rhegmatogenous retinal detachments treated by pars plana vitrectomy with a primary successful or unsuccessful treatment. [file AOS-100-e1600-s001.docx]

| **Supplemental Table S3. Clinical- and treatment characteristics of primary rhegmatogenous retinal detachments treated by pars plana vitrectomy with a primary successful or unsuccessful treatment.** | | | | |
| --- | --- | --- | --- | --- |
|  | **Data expressed as** | **Primary successful (n=845)** | **Primary unsuccessful  (n=27)** | **P** |
| **Age** (years) | *Mean, SD* | 61, 10 | 67, 11 | *0.012* |
| **Tamponade** |  |  |  | 0.213 |
| Air | *% (n=)* | 47.1 (398) | 59.3 (16) |  |
| SF_6_ and C_3_F_8_ gas | *% (n=)* | 52.9 (447) | 40.7 (11) |  |
| **Male gender** | *% (n=)* | 67.6 (571) | 70.4 (19) | 0.76 |
| **Phakic lens** | *% (n=)* | 65.9 (556) | 59.3 (16) | 0.68 |
| **Fovea on** | *% (n=)* | 57.0 (476) | 44.4 (12) | 0.20 |
| **Size retinal detachment** (clock hours) | *Mean, median (Q1-Q3)* | 5.5, 5.0 (4.0-7.0) | 6.7, 6.0 (4.0-9.0) | *0.035* |
| **Area with retinal defects** (clock hours) | *Mean, median (Q1-Q3)* | 2.1, 2.0 (1.0-3.0) | 1.9, 1.0 (1.0-3.0) | 0.62 |
| **Inferior located retinal detachment***** | *% (n=)* | 69.0 (577) | 77.8 (21) | 0.33 |
| **Retinal detachment in 6 o’clock** | *% (n=)* | 30.7 (257) | 59.3 (16) | *0.002* |
| **Inferior located retinal defect**† | *% (n=)* | 38.3 (321) | 33.3 (9) | 0.60 |
| **Retinal defect in 6 o’clock** | *% (n=)* | 10.9 (91) | 18.5 (5) | 0.17 |
| **PVR grade** |  |  |  | *0.016* |
| No PVR or PVR grade A | *% (n=)* | 66.7 (558) | 44.4 (12) |  |
| PVR grade B | *% (n=)* | 33.3 (279) | 55.6 (15) |  |
| **ILM peeling** | *% (n=)* | 3.1 (26) | 11.1 (3) | 0.06 |
| *Statistically significant values are represented in italics. * An inferior located retinal detachment was defined as a detachment involving clock hours 4, 5, 6, 7, and/or 8.* †*An inferior located retinal tear was defined as a defect involving clock hours 4, 5, 6, 7, and/or 8. ILM, inner limiting membrane  PVR, proliferative vitreoretinopathy.* | | | | |
